# Botanicals: A promising approach for controlling cecal coccidiosis in poultry

**DOI:** 10.3389/fvets.2023.1157633

**Published:** 2023-04-25

**Authors:** Zohaib Saeed, Khalid A. Alkheraije

**Affiliations:** ^1^Department of Parasitology, University of Agriculture, Faisalabad, Pakistan; ^2^Department of Veterinary Medicine, College of Agriculture and Veterinary Medicine, Qassim University, Buraidah, Saudi Arabia

**Keywords:** cecal coccidiosis, botanicals, herbal extracts, *Eimeria*, *E. tenella*, poultry, immune response, oxidative stress

## Abstract

Avian species have long struggled with the problem of coccidiosis, a disease that affects various parts of the intestine, including the anterior gut, midgut, and hindgut. Among different types of coccidiosis, cecal coccidiosis is particularly dangerous to avian species. Chickens and turkeys are commercial flocks; thus, their parasites have remained critical due to their economic importance. High rates of mortality and morbidity are observed in both chickens and turkeys due to cecal coccidiosis. Coccidiostats and coccidiocidal chemicals have traditionally been added to feed and water to control coccidiosis. However, after the EU banned their use because of issues of resistance and public health, alternative methods are being explored. Vaccines are also being used, but their efficacy and cost-effectiveness remain as challenges. Researchers are attempting to find alternatives, and among the alternatives, botanicals are a promising choice. Botanicals contain multiple active compounds such as phenolics, saponins, terpenes, sulfur compounds, etc., which can kill sporozoites and oocysts and stop the replication of *Eimeria*. These botanicals are primarily used as anticoccidials due to their antioxidant and immunomodulatory activities. Because of the medicinal properties of botanicals, some commercial products have also been developed. However, further research is needed to confirm their pharmacological effects, mechanisms of action, and methods of concentrated preparation. In this review, an attempt has been made to summarize the plants that have the potential to act as anticoccidials and to explain the mode of action of different compounds found within them.

## Introduction

Coccidiosis is a widespread parasitic disease caused by multiple species of protozoan parasites (1, 2). *Eimeria* spp. are among the most important coccidian parasites, with hundreds of species infecting vertebrates (3, 4). Avian coccidiosis is an absolute intestinal disease characterized by bloody or mucoid diarrhea (5–8). *Eimeria* species are host- and site-specific, and this specificity is below the organ level, i.e., they infect only the intestine, and within the intestine, they have their reserved regions (9, 10). Various species of *Eimeria* show symptoms of the disease from the anterior portion of the intestine to the hindgut, depending on their specified predilection sites (11, 12). Cecal coccidiosis is the most dangerous disease among all types of coccidiosis in avian species (13–15). A high and rapid onset of mortality was only observed in cecal coccidiosis (16, 17). The main causative agent for cecal coccidiosis in broiler chickens is *Eimeria tenella*, which resides in the cecum and causes hemorrhages in it, leading to bloody diarrhea (18–20). Other agents that cause cecal coccidiosis are mentioned in Table 1. This pressing problem highlights the need for scientists to create measures to control coccidiosis, particularly cecal coccidiosis (25, 26).

**Table 1:** The species responsible for cecal coccidiosis in avian species.

**Parasite**	**Host**	**Predilection sites**	**Pathogenicity**	**References**
*E. tenella*	Chicken	Absolute cecal	+++	(21, 22)
*E. brunetti*	Chicken	Ceca, Rectum	++	(22)
*E. necatrix*	Chicken	Ceca, Jejunum, and ileum	+++	(22)
*E. gallopavonis*	Turkey	Ceca, Posterior illum and rectum	+	(23)
*E. adenoeides*	Turkey	Absolute cecal	+++	(24)
*E. meleagridis*	Turkey	Ceca, Small intestine	+	(24)
*E. meleagrimitis*	Turkey	Ceca, Small intestine, rectum	+	(24)

+: Less pathogenic; ++: moderate pathogenic; +++: highly pathogenic.

Currently, multiple drugs are being used in poultry on a daily basis to control coccidiosis (27–29). Due to the acute nature of cecal coccidiosis, preventive measures for controlling this disease are the focus of attention (28–30). Multiple coccidiocidal and coccidiostat drugs are being given in the feed to prevent coccidiosis (31, 32). Anticoccidial drugs target different stages of the Eimeria life cycle, aiming to arrest the parasite at that stage and ultimately control coccidiosis (33).

While chemical anticoccidial drugs have been effective in fighting against coccidiosis, the multiple problems related to this disease have raised doubts about their continued use in the future (34–36).

The primary issue that is being faced with the use of chemical anticoccidials is drug resistance (37–39). Resistance is the ability of the pathogen to escape from the medicine (40, 41). Anticoccidial resistance is a major problem for commercial farmers, as it can result in the wastage of resources and capital on disease control (42, 43). *Eimeria* are resistant to multiple drugs because of multiple mechanisms (21, 27, 44–47). They have developed genetic modifications (48, 49), altered their metabolism, cell membrane permeability, transport channels, and many other ways to escape the drug interaction, leading to the development of resistance (45, 50, 51). Anticoccidial drugs are becoming less effective in treating coccidiosis due to the emergence of increasingly resistant strains of *Eimeria* (52–55).

Resistance is not the only issue with anticoccidial drugs. Multiple scientists also have reported public health issues related to anticoccidial drugs (38, 56, 57). The anticoccidial drug metabolites escape the circulatory system and accumulate in various body parts (58–62). These secondary metabolites are transferred to consumers when they consume meat from animals that have been treated with anticoccidial drugs (58, 63). These drug residues cause several problems and may even be carcinogenic or teratogenic (64–66). Anticoccidial drug residues may cause heart, liver, and kidney failure, leading to death and chronic problems in consumers (67–69). Due to these problems, the European Union has banned the routine use of chemical coccidiostats in feed and allowed a limited amount of use only with the veterinarian's prescription (70–72). These issues and the economics of chemical anticoccidial drugs are forcing researchers to investigate alternatives (36, 73).

Commercially, vaccines are one of the most commonly used alternatives for treating cecal coccidiosis (74–77). Anticoccidial vaccines have been developed to prevent various types of coccidiosis (77). Vaccines are being developed to target various stages of the *Eimeria* and are effective against multiple species of the parasite (56, 78, 79). Anticoccidial vaccines usually use killed parasites or particles of pathogens (80–82). Multiple vaccines, such as Immucox^®^, Livacox,^®^ Coccivac^®^, Hatchpack Cocci-III^®^, Paracox^®^, etc., are being used in routine farming (17, 56, 83). Anticoccidial vaccines can provide immunity, but some issues limit their use (84–87). Vaccine failure is the primary issue observed with anticoccidial vaccines (30, 85, 88). Vaccine failure results in vaccine-induced coccidiosis (89). Moreover, anticoccidial vaccines provide only temporary protection and require frequent repetition, especially in breeders and layer birds (90, 91). The high cost of anticoccidial vaccines limits their widespread use, making them accessible to only breeding and grandparent flocks of chickens (78). These issues necessitate finding a proper alternative to combat cecal coccidiosis.

Several strategies are being explored for the prevention and control of cecal coccidiosis, including the use of organic acids, amino acids, their derivatives, and so on. Botanical substances are also among these proposed alternatives for controlling coccidiosis, as they are gaining attention from scientists for their antioxidant, anti-inflammatory, immunomodulatory, and anti-infectious properties (92–94). Recently, multiple reviews that summarize botanical products that have been proven to have anticoccidial effects have been published (95–101). These reviews have provided valuable insights into how plants can effectively control various forms of coccidiosis. However, there is a need to further investigate the mechanism of action and pharmacological properties of these botanicals. In this review, we have summarized the effective agents of botanicals and their modes of action, as well as the properties that make them beneficial for use against cecal coccidiosis.

## Methodology

This review used Google Scholar (www.scholar.google.com) as the primary search engine. More websites, i.e., ResearchGate (www.researchgate.com) and ScienceDirect (https://www.sciencedirect.com/), and keywords “Cecal coccidiosis;” “*Eimeria* “*tenella*” “*E. tenella;*” “Herbal control of *E. tenella;*” “Use of essential oils for cecal coccidiosis;” “Use of plant extracts for cecal coccidiosis;” “Botanicals for the control of cecal coccidiosis,” “Plants for the control of cecal coccidiosis” were used. The review articles/secondary data were used as a source for the original articles. The data were not quantified, and the statistical comparison was also not performed (102). Table 2 presents the qualitative effects.

**Table 2:** Botanical compounds, their mechanisms of action, and their use for cecal coccidiosis.

**Sr. No**	**Botanical compound**	**Mechanism of action**	**Representative plants/ Products**	**Anticoccidial activities**	**References**
1.	Saponins	They attach to the sterol molecules present in the cell membrane. Stop the metabolism of *Eimeria*. Immunomodulatory and antioxidant activities	Peptasan^®^ (*Acacia concinna*)	Improvement of weight gain and feed intake. Reduction in fecal oocyst count and lesion score.	(101)
			*Y. schidigera*	Improvement in immunomodulatory response and changes in the cecal structure	(103)
			*Quillaja saponaria, Y. schidigera*	Lesion scores reduction and improvement in carcass parameters	(104)
			NorponinXO2 ^®^ (*Y. schidigera* and *Trigonella foenum-graecum*)	The improvement in weight gain and lesion scores	(105)
2.	Tannins	They can penetrate the oocyst wall of *Eimeria*. Deteriorate the cytoplasm and lead to the inactivation of sporulation-inducing enzymes. Potent antioxidants	*Emblica officinalis*	Lesion scores were reduced, and improved weight gain of carcass weight and immune-boosting effects were observed	(106)
			Tannic acid extract product (*Rhus chinensis* gallnuts)	Improved feed conversion ratio, weight gain, and reduced coccidiosis parameters	(107)
			*Pinus radiata*	Showed *in vitro* and *in vivo* anticoccidial activities	(108, 109)
3.	Flavonoids	Prevention of development of *Eimeria* and reduction in the multiplication of merozoites. Strong antioxidants	*Musa paradisiaca*	Reduce the severity of the infection and coccidial parameters	(110)
			*Moringa oleifera*	Lesion scores were reduced, and improved body parameters and immune-boosting effects were observed	(111)
			*Olea europeaea*	The sporulation was infected, and the anticoccidial parameters were reduced in broiler chicks	(111, 112)
			*Ficus racemosa, Syzygium cumini, Cassia fistula*	Antioxidant activities killed the oocyst of *E. tenella*	
4.	Essential oils	Interact membrane sterols. Sporicidal activities by penetration into cell wall Antioxidant and immunomodulatory activities	*M. fragrans*	Stopped sporulation of *E. tenella*, improved body weight gain of organ ratios, and had anticoccidial effects in *in vivo* experiments	(113, 114)
			*P. lentiscus*	Reduction in lesion scores and destruction of oocysts	(115)
			Combination of *O. vulgare* and *Citrus* sp.	Decreased lesion of coccidiosis and improved the body characteristics, improved antibody response.	(116)
			OregoStim^®^ (Based on 5% *Origanum vulgare* essential oil)	Signs of coccidiosis are reduced	(117)
5.	Sulfur compounds	Antioxidants, anti-inflammatory, interact with the cell membrane to destroy cell structure and immunostimulatory agents	Garlicon 40 ^®^ (*Allium* spp.)	*E. acervulina* sporozoites were killed. Antibody response was improved	(118)
			Commercial Herbal Formula (*A. sativum, Satureja hortensis, Chelidonium majus*)	Improved carcass characteristics and decreased oocyst output	(119)
			*A. sativum* and *Z. officinale*	Improved body weight and feed intake reduced the coccidial signs and symptoms	(120)

### Pathology and methods for controlling cecal coccidiosis

It is necessary to identify the points at which coccidiosis can be effectively controlled before reviewing the plants and plant products used to control cecal coccidiosis. Cecal coccidiosis starts with the ingestion of sporulated oocysts of *Eimeria*, which then release sporozoites in the stomach (21, 121, 122). Eimeria releases sporozoites. These sporozoites invade the bird's cecum and penetrate the cecal epithelium (17, 123–126). These sporozoites enter epithelial cells and undergo asexual division named merogony/schizogony, resulting in the formation of schizonts inside the cell (127). Cells containing mature schizonts of *Eimeria* then rupture and release the merozoites (128, 129). These merozoites of *Eimeria* undergo multiple cycles of schizogony, and then, due to unknown reasons, they mature into male and female gametes (micro- and macrogametes) (130, 131). These gametes fuse to form zygotes that develop into unsporulated oocysts, which are then shed in the bird's feces (132, 133).

**Figure 1:**
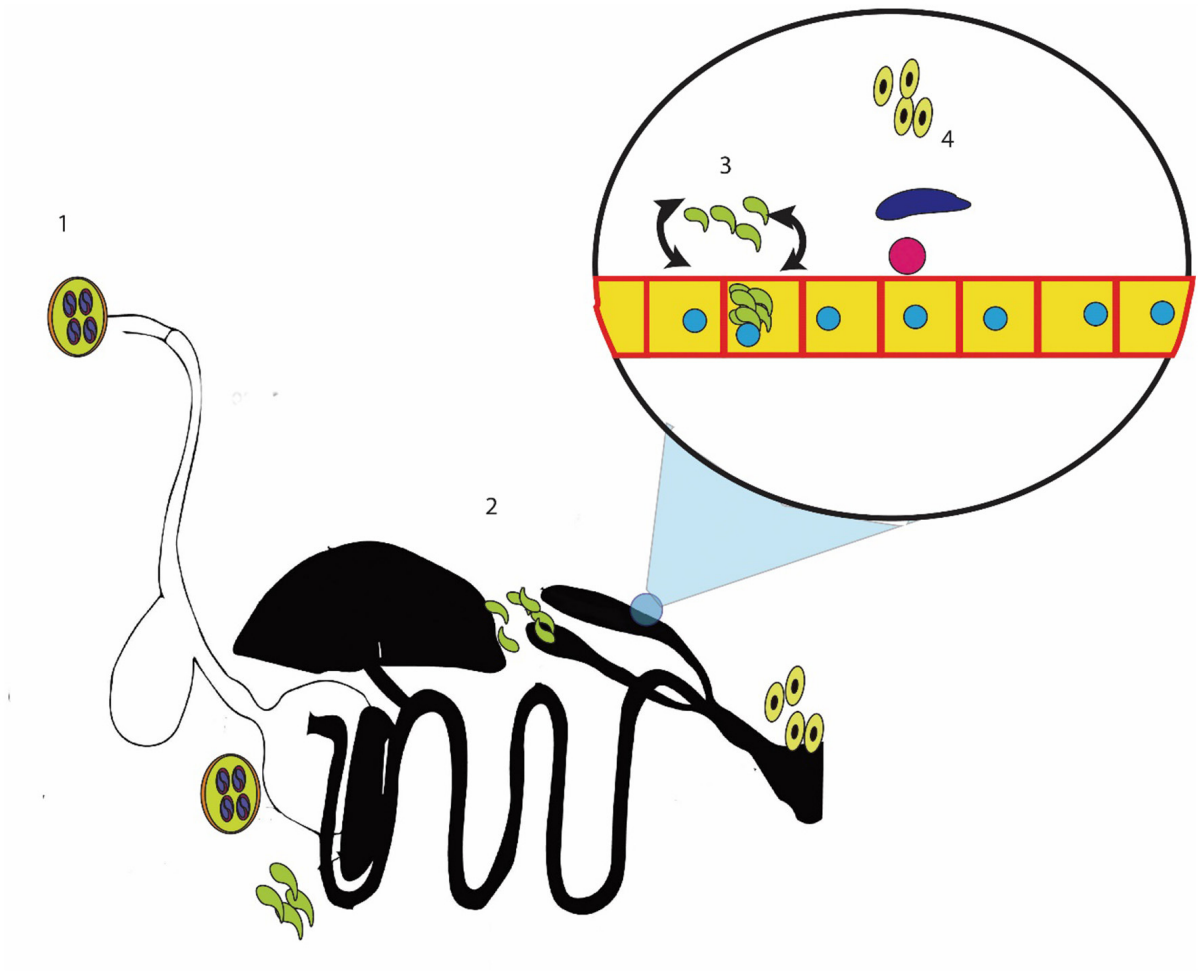
Methods for controlling cecal coccidiosis; 1: killing the sporocyst or reducing the intake of sporocyst; 2: stopping the entry of sporozoites into the cecal epithelium; 3: killing the sporozoites, merozoites, or schizont-containing cells; and 4: controlling the reproductive stage.

The pathogenesis of coccidiosis symptoms arises when the merozoites start infecting the cell and causing the destruction of the epithelium of the cecum (134–136). The epithelium destruction due to schizogony and merozoite invasion induces an immune response (21, 137, 138). This immune response and epithelial destruction result in oxidative stress (139, 140). The reactive oxygen species are produced, and they interact with nearby cells (141–143). These reactive oxygen species extend the damage of coccidiosis to the blood supply to the epithelium, resulting in a blood eruption (144). The repeated cycles of merogony cause extensive damage, leading to bloody diarrhea and death (145). Identifying the different stages of this cycle and their corresponding pathologies provide some points where coccidiosis can be controlled (Figure 1).

First, cecal coccidiosis can be controlled by reducing the intake of sporulated oocysts (21, 146). These sporocysts can be controlled by *in vitro* sporicidal substances, which may kill the sporocysts or stop the sporulation of oocysts (147). The second method of controlling cecal coccidiosis is by reducing the ingestion of sporozoite/merozoite inside the cell (4, 148). Another important method of controlling cecal coccidiosis is reducing the damage caused by *Eimeria*. This can be achieved by stopping oxidative stress and inflammation in the cells and supporting the immune system in its efforts to eliminate *Eimeria* (149, 150). In addition, the number of merogony cycles was reduced and the parasite was forced to enter the sexual phase of the cycle (151, 152). Multiple researchers have explored the use of herbal remedies for controlling cecal coccidiosis, and a few of these remedies have been commercially marketed (119, 153). The active compounds of the plant determine their efficacy in controlling the disease. The active compounds, modes of action, and plants that contain these active ingredients are given in the following sections.

### Botanical compounds for controlling cecal coccidiosis

#### Saponins

Saponins are a group of phytochemicals that are commonly found in many plants (154, 155). They derive their name from their soapy nature, as they can create a foamy texture in aqueous solutions (156–158). Saponins are well known for their antimicrobial, antioxidant, and anticoccidial properties (159–164).

Saponins in botanicals perforate the cell membrane because they interact with cholesterol (158, 165). Cholesterol is the main functional and structural unit of the cell membrane of *Eimeria* (166, 167). In this way, saponins can kill the sporozoites or merozoites of *Eimeria* (55, 168–170). Saponins can directly interact with the sporozoite and stop reproduction (171). Saponins have a high antioxidant capacity, i.e., they can control reactive oxygen species and reduce the pathologies associated with oxidative stress, which is highly present in cecal coccidiosis (172–175).

Botanical saponins have an interesting ability to act as an immune booster, thereby making them suitable candidates for use as vaccine adjuvants (176–178). Multiple researchers have reported that plants containing high amounts of saponins can boost the immune system by affecting the immune organ maturation and increasing the antibody levels in the body, providing better defense against cecal coccidiosis (164, 179–182). Plants containing saponins also function as astringents, i.e., they reduce surface tension inside the body and help nutrients enter the cells (183, 184). Due to these activities, multiple saponin-containing plants have been used to control coccidiosis (101, 105, 119, 185–190).

#### Tannins

**Figure 2:**
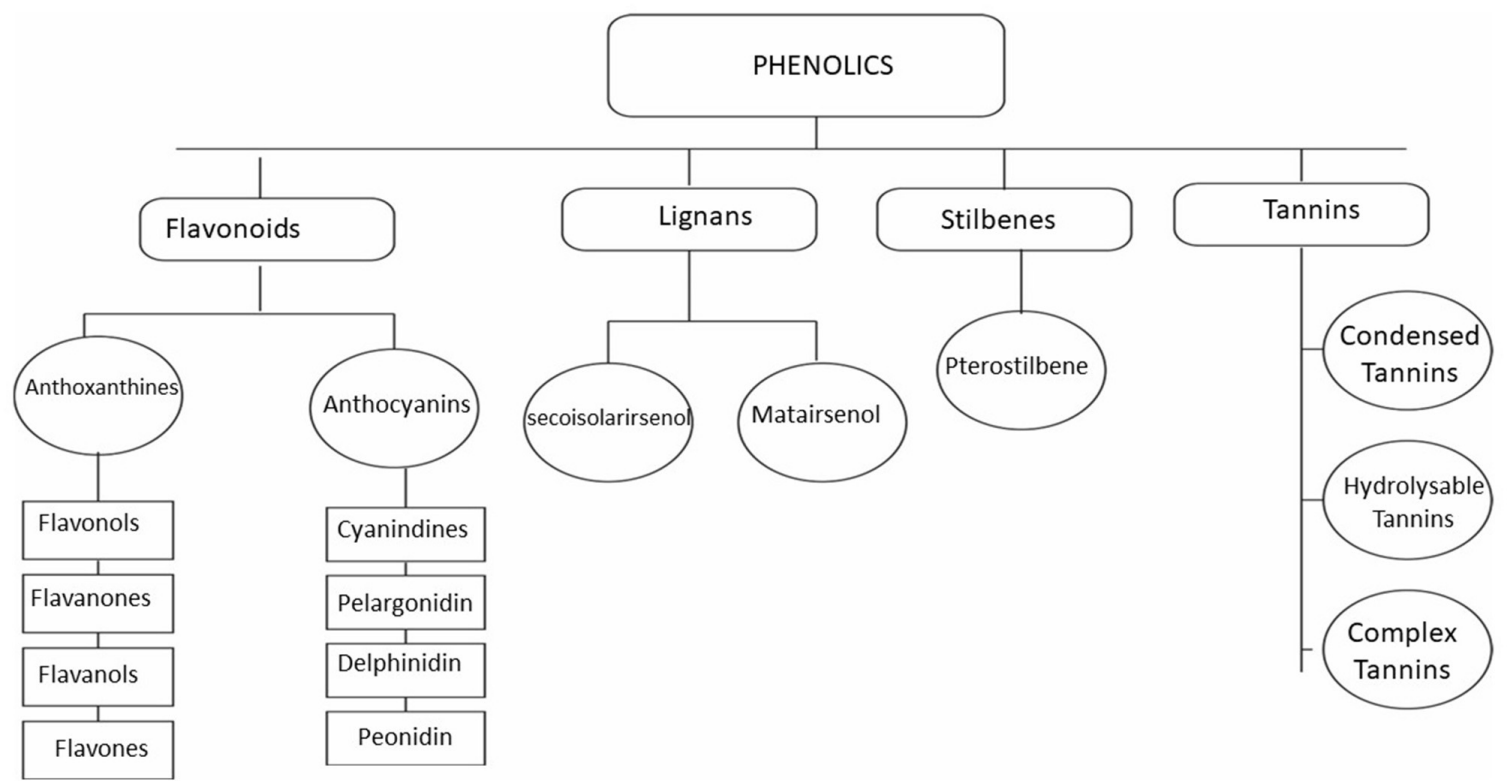
Classification of phenolics.

Tannins, or tannic acids, are a ubiquitous class of molecules that occur naturally in plants as defense compounds (191–193). Tannins are classified as phenolic compounds (Figure 2), and they contain various functional groups (194–196). They act as pesticides in plants, protecting them from invading pests and microbes (197, 198). They help regulate plant growth by protecting them from infectious agents (199–203). Tannins can coagulate bacterial cell walls by affecting their peptides (196, 204, 205). Peptide units also make the oocyst wall of *Eimeria* (206, 207); thus, they perforate the oocyst wall of *Eimeria* oocysts and denature them (188). Tannins can also protect the epithelium cells from damage and maintain the integrity of nucleic acid during microbial infections (208–210). Tannins also show immunomodulatory activities and potentiate the immune response, helping control infections (211–214). Tannins are also antioxidants and reduce oxidative stress, a condition commonly associated with numerous diseases, especially cecal coccidiosis (21, 215). Numerous tanniferous plants have been used to control coccidiosis (30, 216–222). The efficacy of these plants in controlling cecal coccidiosis can be attributed to their immunomodulatory, antioxidant, and direct anticoccidial activities (187, 222–225).

#### Flavonoids

Flavonoids are a diverse and broad class of plant phenolic compounds (Figure 2) (226–228) and constitute one of the most prevalent phenolic compounds (229–232). They are further divided into flavonols, flavanols, flavanones, flavones, etc., as described in Figure 2 (233–235). They have multiple modes of action depending on the type and class of flavonoids (236–238). Flavonoids have the potential to control coccidiosis due to their well-known oxidative stress reduction activities (239–241). Flavonoids can also penetrate the cell membranes and cause sporozoite and oocyst death (171, 242). Multiple plants, such as, *Moringa oliefera* and *Syzygium aromaticum*, with active flavonoids have anticoccidial activity (170, 188, 240, 243–245).

#### Essential oils

Essential oils are volatile, short-chain, lipophilic portions of plants that can be extracted using hydro or steam distillation techniques (246–249). They usually contain terpenes, terpene derivatives, aldehydes, and other compounds (250–252). Essential oils are highly active antioxidants and immunomodulators (253–257). These oils can kill the oocyst of *Eimeria* (15, 258–260) and stop sporulation by penetrating the walls of the *Eimeria* oocyst (147, 261–263). Essential oils can also help reduce coccidial signs and symptoms because of their direct and indirect anticoccidial activities (264). Essential oils have been widely used to control coccidiosis, especially the cecal kind (15, 116, 117, 259, 262, 265–272).

#### Vitamins and minerals

Vitamins and minerals are the crucial micronutrients needed by the body to regulate multiple functions and the metabolism of the body (273, 274). Vitamins and minerals are naturally present in plants. They play an important role in many metabolic reactions and help the body maintain growth. Similarly, multiple minerals act as co-factors of the enzymes and have been used to control coccidiosis (259). Vitamins and minerals act as antioxidants and immune stimulators, helping control cecal coccidiosis (275–277). Vitamins and minerals, i.e., vitamin E, selenium (278, 279), vitamin K (280), vitamin A, vitamin D (281, 282), etc., have been shown to be effective in reducing the signs and symptoms of coccidiosis in various multiple experiments.

#### Sulfur compounds

Sulfur compounds are usually present in the garlic family (*Allium spp*.) and cannabis (283, 284). Allicin, diallyl disulfide, propyl thiosulfinate oxide, allyl methyl thiosulfate, etc., are commonly found sulfur compounds in plants (285, 286). They have been shown to have high antimicrobial, antioxidant, and anti-aging properties in multiple experiments (287, 288). They have shown multiple bioactivities in many experiments. Sulfur compounds can potentially kill *Eimeria* by destroying the sporozoites (21). Garlic in multiple forms, i.e., essential oils, extracts, and powders, has been used to treat coccidiosis, and it has shown anticoccidial activity due to these compounds (89, 120, 124, 217, 289, 290).

### Pharmacological interactions of botanicals

In a single plant, hundreds of compounds may show multiple activities (291, 292). In multiple experiments, more than one plant was used to control cecal coccidiosis (119, 120, 293). When we added multiple bioactive compounds, there was a possibility of multiple pharmacological interactions occurring among them (294). These interactions may be synergistic, additive, antagonistic, etc. Thus, it is necessary to determine whether these plant products exhibit synergistic or additive effects or if they demonstrate antagonistic properties (295–297). Several experiments have demonstrated that botanical compounds can exhibit additive and synergistic effects against caecal coccidiosis (56, 244, 298). Due to the presence of multiple compounds, plants can employ multiple mechanisms to control *Eimeria*, thereby reducing the likelihood of drug resistance (27, 299). Although reports suggest that interactions exhibit synergistic or additive effects, further research is needed to explore their potential antagonistic effects.

## Conclusion

Studies have shown that plants contain multiple types of botanical compounds that differ in their quantities and ratios. These compounds have demonstrated antioxidant and anticoccidial properties against avian caecal coccidiosis. Phenolics, saponins, essential oils (including terpenes and derivatives), sulfur compounds, etc., have been proven to exhibit anticoccidial effects through diverse mechanisms of action. Moreover, it should be noted that these plant compounds may possess antinutritional properties, which can lead to reduced feed intake, growth inhibition, and adverse effects on body growth. However, studies on their potential toxicity are lacking with respect to coccidiosis. Before considering their therapeutic use, it is imperative to investigate their toxicological profiles. Furthermore, further research is needed to evaluate their pharmacological interactions within the body.

## Author contributions

ZS wrote the article and created the illustrations. KA managed the references and edited the manuscript. All authors contributed to the article and approved the submitted version.
